# Plant Sterol Clustering Correlates with Membrane Microdomains as Revealed by Optical and Computational Microscopy

**DOI:** 10.3390/membranes11100747

**Published:** 2021-09-29

**Authors:** Ling Tang, Yang Li, Cheng Zhong, Xin Deng, Xiaohua Wang

**Affiliations:** 1 Key Laboratory of Plant Resources, Institute of Botany, Chinese Academy of Sciences, Beijing 100093, China; tangling181@mails.ucas.edu.cn (L.T.); liyang184@mails.ucas.edu.cn (Y.L.); deng@ibcas.ac.cn (X.D.); 2 College of Life Sciences, University of Chinese Academy of Sciences, Beijing 100049, China; 3 Hubei Key Lab on Organic and Polymeric Optoelectronic Materials, Department of Chemistry, Wuhan University, Wuhan 430072, China; zhongcheng@whu.edu.cn

**Keywords:** FLIM, computational microscopy, phase separation, microdomain, phytosterol

## Abstract

Local inhomogeneities in lipid composition play a crucial role in the regulation of signal transduction and membrane traffic. This is particularly the case for plant plasma membrane, which is enriched in specific lipids, such as free and conjugated forms of phytosterols and typical phytosphingolipids. Nevertheless, most evidence for microdomains in cells remains indirect, and the nature of membrane inhomogeneities has been difficult to characterize. We used a new push–pull pyrene probe and fluorescence lifetime imaging microscopy (FLIM) combined with all-atom multiscale molecular dynamics simulations to provide a detailed view on the interaction between phospholipids and phytosterol and the effect of modulating cellular phytosterols on membrane-associated microdomains and phase separation formation. Our understanding of the organization principles of biomembranes is limited mainly by the challenge to measure distributions and interactions of lipids and proteins within the complex environment of living cells. Comparing phospholipids/phytosterol compositions typical of liquid-disordered (Ld) and liquid-ordered (Lo) domains, we furthermore show that phytosterols play crucial roles in membrane homeostasis. The simulation work highlights how state-of-the-art modeling alleviates some of the prior concerns and how unrefuted discoveries can be made through a computational microscope. Altogether, our results support the role of phytosterols in the lateral structuring of the PM of plant cells and suggest that they are key compounds for the formation of plant PM microdomains and the lipid-ordered phase.

## 1. Introduction

Plasma membranes (PMs) are heterogeneous material assemblies in which the preferential association of certain phospholipids, sterols and proteins can lead to the formation of microdomains or nanodomains, so-called lipid rafts, with a diameter of 10–100 nm [1]. Sterols play a fundamental role in the formation of lipid rafts in the plasma membrane, which in turn are involved in many intra- and inter-cellular processes, including membrane fluidity, stress responses, compartmentalizing signal transduction and membrane trafficking events [2,3,4,5,6,7]. The plasma membranes of higher plant cells contain a complex mixture of sterols [8], unlike those in mammals and fungi, where cholesterol and ergosterol, respectively, are the only type of sterol form. Strikingly, sterols can represent up to 20% of all lipids in plant membranes and 30–50% in animal membranes. The phytosterols (that is, C-24 alkylsterols) stigmasterol, sitosterol and campesterol are the most abundant sterols in the plant kingdom. Besides their major importance as membrane components, phytosterols serve as precursors for brassinosteroids, signaling molecules involved in growth and development [9]. In model membranes, large amounts of cholesterol induce the lateral formation of coexisting domains that are described as a phase separation between liquid-ordered (defined as lipid rafts) and liquid-disordered phases [10,11]. Brewster angle microscopy (BAM) is a powerful technique that provides a quantitative structural analysis of Langmuir monolayers. BAM imaging along with Langmuir techniques or X-ray reflectometry have also been widely used to study domain morphology and lateral phase separation in lipid monolayers and interfaces [12,13,14,15,16]. However, apart from cholesterol, the investigations on the other membrane sterols are not comprehensive in nature based on BAM approaches. Importantly, total internal reflection fluorescent microscopy (TIRFM) and super-resolution optical microscopy have supported the lipid raft hypothesis by providing direct evidence of membrane microdomains in vivo [4,17,18,19]. Due to instability (half-life 10–20 ms, [17]) and size (about 100 nm, [20]), microdomains are still rarely annotated using microscopic visualization due to the diffraction limits for optical approaches.

Membrane microdomains, sometimes referred to as lipid rafts, are proposed to be liquid-ordered phase domains. With sterol and sphingolipid in the membrane, the domains may be in liquid-ordered and liquid-disordered phases. Liquid–liquid phase separation (LLPS) of biomolecules has emerged as an important platform that contributes to cellular organization, cell signaling, molecular trafficking and pathogen response [21,22,23]. LLPS also occurs at membranes, where both lipids and membrane-associated proteins can de-mix to form phase-separated compartments [24,25,26]. Very recently, many efforts have been made to take advantage of the sensitivity of fluorescence lifetime to local properties of lipid bilayers for studying lipid domains [27,28,29,30]. The lifetime of membrane staining with phase-sensitive probes, Laurdan and di-4-ANEPPDHQ, was higher in the Lo phase than that of the Ld phase [31,32]. Using a new solvatochromic push–pull fluorophore based on pyrene, Niko et al. could distinguish the Lo and Ld phases in giant unilamellar vesicles (GUVs) and HeLa cells [13]. However, these methods are still typically limited to measurements in plant cell membranes. In the present study, we used fluorescence lifetime imaging microscopy in conjunction with the solvatochromic fluorophore, referred to as the push–pull probe, to directly visualize the lipid order domain and phase separation in living plant cells.

It is not straightforward to achieve a molecular look at a cell membrane using experimental data, since the accuracy limitations of the measurements and the interpretation of the involved models complicate the picture. One way to study the dynamic behavior of these molecules at near-atomic resolution is through the use of molecular dynamics (MD) simulations, which provide reasonable and reliable results compared to experimental data [33,34,35,36]. By using such simulations as a “computational microscope” [37,38], we are able to obtain information on their single molecule dynamics, interactions with other lipids and with membrane proteins, thus characterizing emergent properties and aiding the interpretation of experimental data. Sanchez et al. demonstrated that MβCD preferentially removes cholesterol from liquid-disordered (Ld) instead of liquid-ordered (Lo) membrane domains [39]. More recently, Khuntawee et al. have shown that βCD passively diffuses into the lipid bilayer by pointing its open secondary rim toward the lipid polar groups with hydrogen bond formation [40]. Although sterol–lipid interactions have long been recognized as major determinants of membrane structure and homeostasis in living cells, the nature of phytosterol–phospholipid interaction and how the phytosterols modulate the mechanical properties of lipid membranes are still not completely resolved. Therefore, deeper experimental studies and all-atom MD simulations to understand how phytosterols are able to create raft domains in plant plasma membranes, even in model membranes, are required. Here, we performed fluorescence lifetime imaging (FLIM) analysis in living plant cells and all-atom long-timescale MD simulations with regard to structural properties, such as lipid area, thickness and order degree, as well as dynamic properties, including diffusion of lipids in the mixture lipid bilayer for higher plant cells. Collectively, the experimental and computational microscopy study here enables a comprehensive examination of phytosterol characteristics and the effects on the structural properties of membrane domain formation and phase separation.

## 2. Materials and Methods

### 2.1. Plant Materials

*Arabidopsis thaliana* ecotype Col-0 and *smt2smt3* mutant seeds were firstly surface sterilized in 70% ethanol for 2 min and 5% (*w*/*v*) NaClO for 15 min. After washing three times with sterilized distilled water, the seeds were grown vertically on 1/2 MS medium solidified with 1% agar at a pH of 5.8. The seedlings were grown at 22 °C for 4 days under a 16 h/8 h light/dark cycle on 1/2 MS solid medium plates.

### 2.2. Plant Cell Labeling and Drug Treatment

The PA probe was kindly provided by Dr. Konishi [13] and dissolved in DMSO at 100 μM stock. The PA stock solutions were diluted with liquid 1/2 MS medium for use, and the final DMSO concentration was ≤0.1% (*v*/*v*), and equivalent volumes of DMSO were added to the controls. The plant cells were stained by the PA probe in a final concentration of 100 nM for 5 min before imaging under a laser scanning confocal or fluorescence lifetime imaging microscope. The phytosterol depletion reagent methyl-β-cyclodextrin (MβCD, Sigma-Aldrich, MO, USA) was dissolved in deionized water to yield a 200 mM stock solution. The 4-d-old Col-0 seedlings were incubated in 1/2 liquid MS medium containing 5mM MβCD for 30 min before labeling with 100 nM PA probe.

### 2.3. Time-Resolved Fluorescence Spectroscopy and Image Analysis

Fluorescence microscopy imaging of PA-labeled plant cells was performed using a laser scanning setup based on an Olympus IX70 inverted microscope with an Olympus 60 × 1.2 NA water immersion objective. The PA probe was excited using the 488 nm laser line of an argon ion laser. The emission fluorescence was detected at two spectral ranges: 500–550 nm (blue channel) and 580–700 nm (red channel). Image analysis was carried out by ImageJ 1.80 software (https://imagej.nih.gov/ij/download.html, accessed on 10 June 2021). The ratiometric and generalized polarization (GP) images were generated by a previously described protocol [41] that divides the image of the red channel by that of the blue channel.

### 2.4. Fluorescence Lifetime Imaging and Data Analysis

The FLIM system (LSM kit, Picoquant, Berlin, Germany) was incorporated into the Olympus IX70 and consisted of a pulsed diode laser (485 nm, FWHM = 83 ps, 20 MHz) and was equipped with a time-correlated single-photon counting module from PicoQuant (Berlin, Germany). The emission signal was collected through a band-pass emission filter centered at 510 nm (FWHM = 20 nm) using single-photon avalanche diode detectors (SPADs, PicoQuant, Berlin, Germany). Fluorescence lifetime measurements were acquired using software (SymPhoTime 64 version 2.0, PicoQuant) that controlled both the TCSPC system and the scanner system. Acquisition times were adjusted to achieve 1000 photons per pixel (with a typical acquisition time of 3 min). FLIM data were analyzed with a binning of 2 using the FLIMfit v5.1.1 software (https://flimfit.org/, accessed on 15 January 2021), which uses a separable nonlinear least square algorithm to recover the lifetimes from the fluorescence decays [42]. The whole-cell lifetime intensity decays were fitted with the mono-exponential decay function deconvoluted with the instrument response function (IRF) to generate FLIM images showing the apparent lifetime of each pixel. The reduced chi-squared (χ2) value from the mono-exponential function is sufficient to describe the lifetime data in the present study.

### 2.5. Atomistic Molecular Dynamics Simulation and Quantum Chemistry Calculation

The mixture lipid bilayers were generated using the CHARMM graphical user interface (CHARMM-GUI version 1.9) Membrane Builder [43]. The main simulation system for each model contained 768 lipids evenly distributed in two leaflets with neutralizing ions and was fully hydrated using TIP3P water. GROMACS version 2019.3 [44] was used for all atomistic molecular dynamics simulations, with the CHARMM36 (CGenFF) force field parameters [45]. The simulations were performed at a temperature of 298 K using a Berendsen thermostat with τp = 0.1 ps. A constant pressure of 1 bar was maintained with an isotropic coupling constant τP = 1.0 ps and compressibility = 4.5 × 10^−5^ bar^−1^. The integration time step was 2 fs. Periodic boundary conditions and the particle-mesh Ewald algorithm [46] were used to account for the long-range electrostatic effects. The LINCS method was used to constrain bond lengths. Coordinates were saved every 2 ps for subsequent analysis. The structure optimizations of the PA probe were performed using the Gaussian 16 software [47] and the density functional (DFT) method cam-B3LYP with the def2svp basis-set. Based on these optimized geometries, the HOMO–LUMO gaps were identified using Multiwfn [48] and visualized with VMD [49].

### 2.6. Molecular Dynamics Simulation Analysis

Partial density, membrane thickness and lipid ordering plots were calculated using analysis soft packages developed by the Luca group in IBCP (CNRS), France. All landscapes were calculated using g_thickness, g_ordercg and g_mydensity software [50], freely available from http://perso.ibcp.fr/luca.monticelli (accessed on 20 March 2021). The binding free energy was calculated based on the molecular mechanics Poisson–Boltzmann surface area (MM-PBSA) method [51] with the g_mmpbsa code. The binding free energies during lipid–lipid interactions were analyzed during the equilibrium phase by taking 1000 snapshots from the last 40–50 ns at an interval of 10 ps from the MD trajectories. The APL@Voro program [52] was used to obtain the Voronoi diagrams of the individual area per lipid and lipid thickness values in the mixed bilayers. To calculate the projected area per lipid, the coordinates of the key atoms of different lipids are projected onto a plane, and a Voronoi approach is calculated using these projected coordinates. The MD trajectories were visualized, and all of the snapshots in this article were presented using the VMD software [49].

### 2.7. Statistical Analyses

Both the experimental and simulation data reported represent at least two independent replicates. Where not mentioned, the data are shown as mean values ± standard errors. Statistical analyses were performed using R v.4.0.3 (www.R-project.org, accessed on 10 July 2021). Mean comparisons were calculated by Student’s t-test with P-values, and data sizes are indicated in the figure legends. All data were graphed using R v.4.0.3.

## 3. Results

### 3.1. Push–Pull PA Reports Lipid Order-Disorder in Plant Cell Membranes

**Figure 1:**
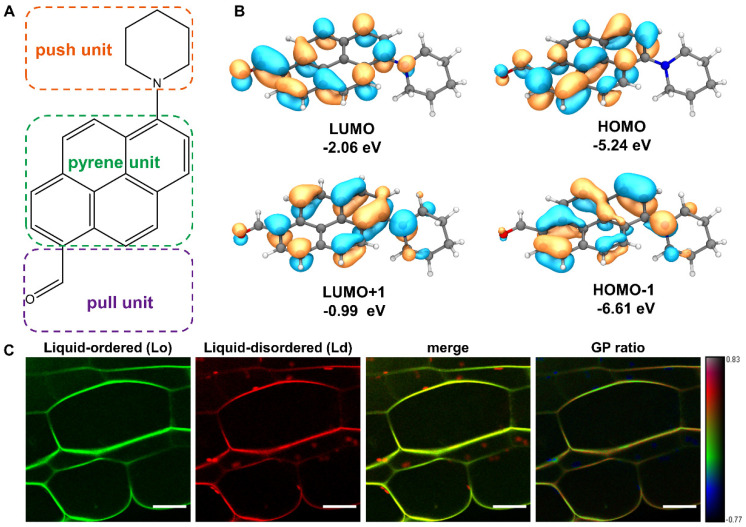
Structure of PA probe and the spectral GP imaging of living plant cells. (**A**) Chemical structure of the new push–pull dye based on the pyrene backbone. (**B**) Energy diagrams and orbital distributions (HOMO–LUMO gap) of PA molecule calculated at cam-B3LYP/def2svp level of theory. Orange and blue color distributions represent a positive and negative phase in molecular orbital wave function, respectively. (**C**) Fluorescence intensity (green and red channels) and merged images of living Arabidopsis root cells stained with PA. The rightmost pseudo-color image represents the ratio of the long- to-short wavelength emission channels (580–700 nm to 500–550 nm). The GP scale used to pseudo-color the intensity image can differentiate liquid-ordered and -disordered domains coexisting in plasma membranes. Scale bar, 20 μm.

PA is a newly developed solvatochromic push–pull fluorescent probe based on pyrene (Figure 1A). The DFT calculations confirmed that PA is a typical donor-π-bridge-acceptor (D-π-A) chromophore. Both the highest occupied molecular orbital (HOMO) and the lowest unoccupied molecular orbital (LUMO) are partially localized on the pyrene ring attached to the carbonyl group (Figure 1B). The calculated EHOMO and EHOMO-1 were in the range −5.24 to −6.61 eV, while ELUMO+1 was −2.06 to −0.99 eV, which resulted in ΔE_HOMO−LUMO_ = 3.18 eV. As can be seen in Figure 1B, the HOMO–LUMO electronic density is quite similar, and the corresponding small HOMO–LUMO gap can also be seen, thus validating that PA is a very sensitive and easily excited fluorescent probe. To understand the plasma membrane dynamics and the lipid microdomain organization, we detected the fluorescence signals in *Arabidopsis* root cells stained with PA. Already after 5 min of incubation with 100 nM PA, fluorescence was observed all over the root cell membrane. The two fluorescent emission channels, the green channel (500–550 nm) and the red channel (580–700 nm), are correlated to the liquid-ordered (Lo) phase and the liquid-disordered (Ld) phase, respectively (Figure 1C). The spectroscopic response of PA to the degree of lipid order in the plant cells was similar to those obtained in DOPC vesicles (Ld phase) and SM/Chol vesicles (Lo phase) with a maximum emission at around 581 and 528 nm, respectively [13]. As can be seen in the last panel of Figure 1C, PA distinguished the Lo and Ld phases well through strong changes in the generalized polarization (GP) ratio signal. Notably, the chloroplasts inside the cell can also be labeled by PA with a remarkably low GP ratio. Collectively, the PA probe reveals that the plant plasma membranes are mainly presented by the Lo phase, while the intracellular chloroplasts are much less ordered being close to the Ld phase.

### 3.2. Fluorescence Lifetime of PA in Living Plant Cell Membrane

**Figure 2:**
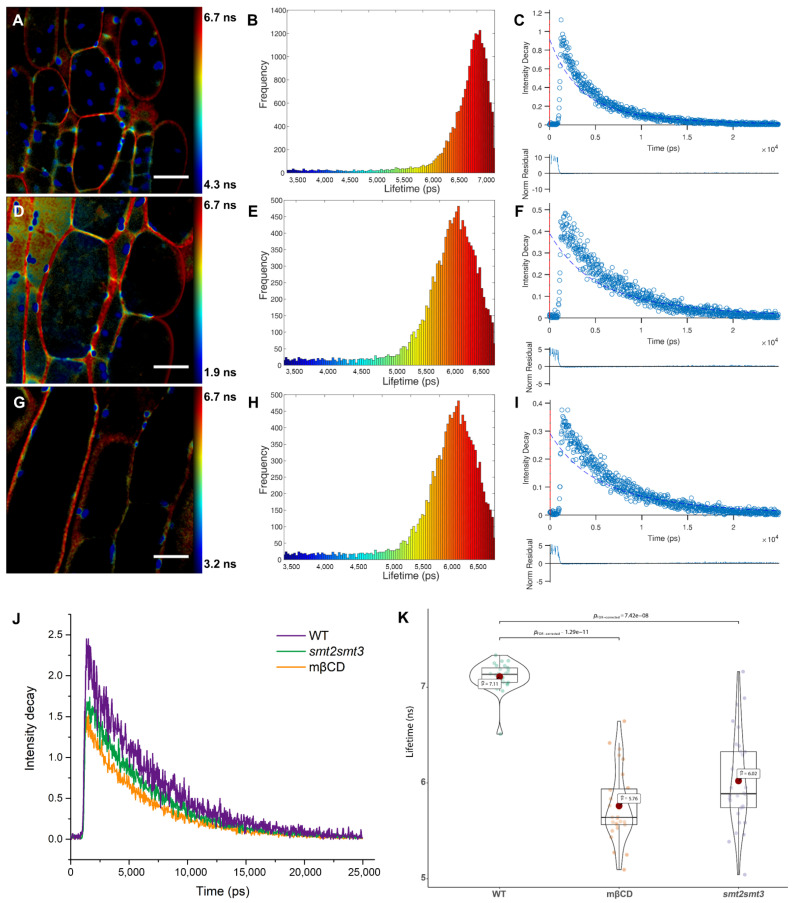
Fluorescence lifetimes of PA in plasma membrane with different phytosterol content. (**A**–**C**) FLIM images (**A**), lifetime distribution (**B**) and an example fit (**C**) for the fluorescence decays (top) and normalized residuals (bottom) of intact plant cells. (**D**,**E**) FLIM images (**D**), lifetime distribution (**E**) and an example fit (**F**) for the fluorescence decays (top) and normalized residuals (bottom) of *smt2smt3*-mutant cells. (**G**–**I**) FLIM images (**G**), lifetime distribution (**H**) and an example fit (**I**) for the fluorescence decays (top) and normalized residuals (bottom) of phytosterol-depleted cells. The colors of the pixels describe the values of the lifetime components according to the color scale on the right panel. (**J**) Global analysis of fluorescence intensity decay of intact (WT) and phytosterol-depleted cells (*smt2smt3* and mβCD treated wild-type lines). (**K**) Quantitative analysis and statistical comparison of average lifetimes in intact and phytosterol-depleted cells (n = 20). Scale bar, 20 μm.

To characterize the observed lipid order in the PA-labeled plant plasma membrane, we used fluorescence lifetime imaging (FLIM) approaches. First, we measured the average fluorescence lifetime in intact (wild type) and phytosterol-depleted cells using the time-correlated single-photon counting (TCSPC) technique. Phytosterol-depleted cells were obtained by use of *smt2smt3* mutant or incubation of the wild-type seedlings with 5 mM MβCD for 30 min. We analyzed the FLIM images’ serials by employing a single-decay-component model. As shown in Figure 2A,D,G the FLIM images of intact and phytosterol-depleted cells labeled with PA showed a remarkable heterogeneity in their pseudo-color distribution, indicating that phase separation could be perceived, especially in the plasma membrane. We further carried out a detailed analysis of the fluorescence lifetimes of PA in cells with different phytosterol compositions through constructing histograms of the distribution and variability of lifetimes across the whole images (Figure 2B,E,H). The lifetime distributions were centered at 7.11 ± 0.36, 6.02 ± 0.23, 5.76 ± 0.35 ns for intact, *smt2smt3* and phytosterol-depleted cells, respectively (Figure 2J). The fluorescence decays measured for PA in cells with different phytosterol are shown in Figure 2K. The fluorescence decays are mono-exponential (Figure 2C,F,I), and the fluorescence lifetime varies markedly among the plant cells in relation to the phytosterol content. In the FLIM images of cells in different seedlings stained with PA, the cytoplasm appeared in green-to-blue (with lifetime ~4 ns). By contrast, cell plasma membrane appeared in yellow-red pseudo-color, indicating a much longer lifetime (~6 ns). Thus, in line with our ratiometric data (Figure 1C), cell plasma membrane is much more ordered (Lo phase) than intracellular membranes. This means that the intracellular membranes are close to the Ld phase. As these two phases could not be clearly separated in the FLIM images (Figure 2A,D,G), this suggests that the Lo-like and Ld-like domains are smaller than the ~200 nm resolution limit of our fluorescence lifetime microscope and/or that the domains are highly dynamic and, thus, cannot be resolved in this temporal scale (~1 s) of the FLIM measurements.

### 3.3. All-Atom System Mimicking the Plant Plasma Membrane

**Figure 3:**
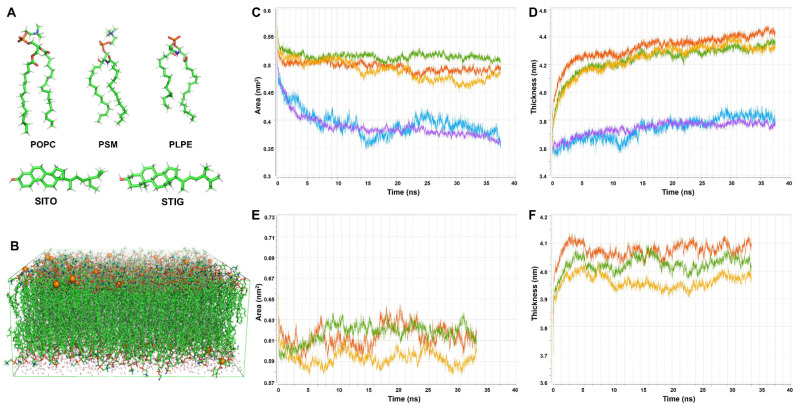
The composition of the model plasma membrane and the lipid dynamics changes over time. (**A**) The structure of the POPC, PSM, PLPE, SITO and STIG lipid molecules was examined in this study. (**B**) A snapshot of a typical configuration of a mixed lipid bilayer in water. The lipids are shown in green lines, water is shown in red, and the potassium ions are shown in orange spheres. (**C**,**D**) The time evolution of the area per lipids and membrane thickness in plasma membrane containing phytosterols over time, respectively. (**E**,**F**) The time evolution of the area per lipids and membrane thickness in plasma membrane containing phytosterols over time, respectively. Green plot: POPC. Red plot: PSM. Orange plot: PLPE. Blue plot: STIG. Purple plot: SITO.

In order to explore the behavior in super temporal–spatial scales of complex lipid bilayers, all-atom lipid bilayer models containing 768 lipid molecules were generated applying CHARMM-GUI. These were derived from an initial model bilayer containing a POPC, PSM, PLPE, PLPI, STIG and SITO (Figure 3A) mixture membrane (molar ratio 26:13:28:5:6:22 [53,54]) with dimensions of 16 × 16 nm (Figure 3B). This membrane composition and distribution mimic a plant plasma membrane. A corresponding model membrane without phytosterols (STIG and SITO) was additionally constructed and simulated as a phytosterol-depleted treatment. All the MD simulations (100 ns simulations for each of the systems) were performed with CHARMM36 all-atom force field to represent an accurate membrane environment. Area per lipid (APL) is another standard way to determine when a membrane simulation is physically sensible. Figure 3C,E show APL versus time for a representative production run of these model plasma membranes. The membrane thickness is equilibrated after the first 10 ns (dashed gray line), and indeed, the thickness over the last 40 ns never drifts far from the average thickness of 4.2 ± 0.3 nm calculated over the same time (Figure 3D,F).

### 3.4. Spontaneous Membrane Insertion of PA

**Figure 4:**
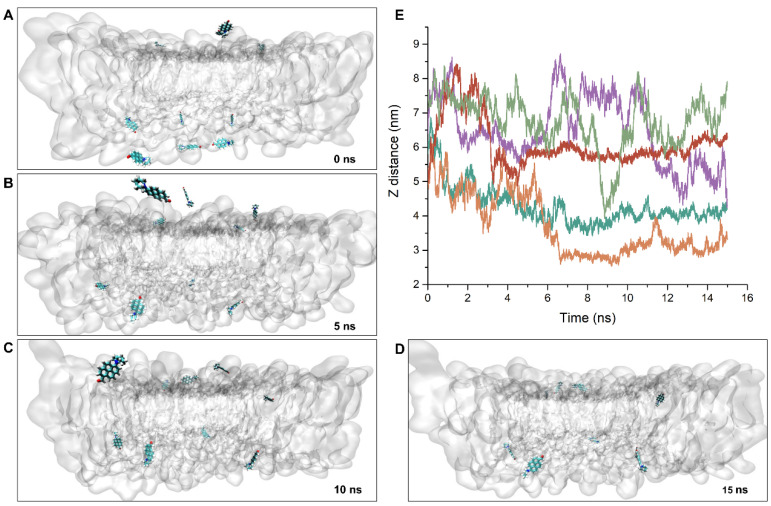
The snapshots of the partition and insertion kinetics of PA into lipid bilayer at different simulation times. (**A**–**D**) The 16 ns MD simulation was based on random and passive distribution of probe molecules in water phase above or below the bilayer. Ten PA molecules were sampled to ensure better MD statistics. For clarity, the lipid molecules are shown in transparent representation. (**E**) The corresponding plots of the distance between geometrical center of PA and the bilayer midplane. The plots showed that the PA molecules diffuse randomly, independent of each other, and the stable insertion is spontaneous with the shortest time at 7 ns.

Considering PA is suitable for studying membrane dynamics and phase separation, it is of interest to study membrane–fluorophore interactions and probe distribution in lipid bilayers. Figure 4A–D show that during the first 15 ns period of the 10 PA molecules, which were initially placed at different positions in the vicinity of the bilayer, they moved to the bilayer interface. We found that when the PA molecules reached the bilayer interface, their further movement slowed down (Supplementary Video S1). At the end of the 15 ns of the MD simulation, spontaneous membrane insertion events were observed in both sides of the lipid bilayers (Figure 4D). Figure 4E shows the z position versus time between the geometrical center of PA and the bilayer midplane. The presence of the pyridine moiety appears to oppose the formation of initial PA membrane contact, and the fluorophores are hence repelled away from the membrane surface (Figure 4E, olive green trajectory). In contrast, the carbonyl moiety favors the residence and location of PA in the bilayer center. At the end of the 15 ns MD simulation, 4 out of the 10 PA molecules were observed to have stable and full insertion into the middle of the lipid bilayer.

### 3.5. Phytosterols Play Crucial Roles in Homeostasis of Membrane Area and Thickness

**Figure 5:**
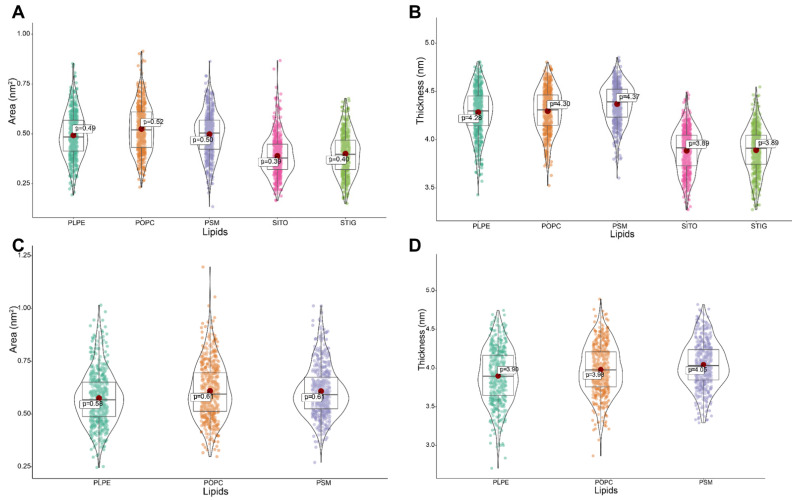
Phytosterols’ effect on lipid area and thickness in model lipid bilayer. (**A**,**B**) Quantitative analysis of average area and thickness of lipids in model membranes containing phytosterols. (**C**,**D**) Quantitative analysis of average area and thickness of lipids in model membranes not containing phytosterols.

To characterize the effect of phytosterols on plant membrane structure, we calculated the area and thickness of the lipids in the model membranes defined as the average distance of P–P or O-O (phytosterols) atoms in the lipid head groups (Figure 3A). Large variations were observed in the lipid area and thickness in the model membranes with and without phytosterols (Table 1). For the model membrane with phytosterols, we obtained the average area of 0.49, 0.52, 0.50, 0.39 and 0.40 nm^2^ for PLPE, POPC, PSM, SITO and STIG, respectively. In contrast, an increase in lipid area with an average value of 0.58, 0.61 and 0.61 nm^2^ for PLPE, POPC and PSM was observed, followed by the lack of phytosterols (Table 1 and Figure 5A,C). Our data also showed that the lipids became more densely packed in the membrane containing phytosterols, and the membrane has a larger thickness value than that of the phytosterol-free membrane. (Figure 5B). On the contrary, the lack of phytosterols encourages loose packing and decreases chain order; the membrane seems to possess a tendency to reduce the lipid thickness (Figure 5D).

**Figure 6:**
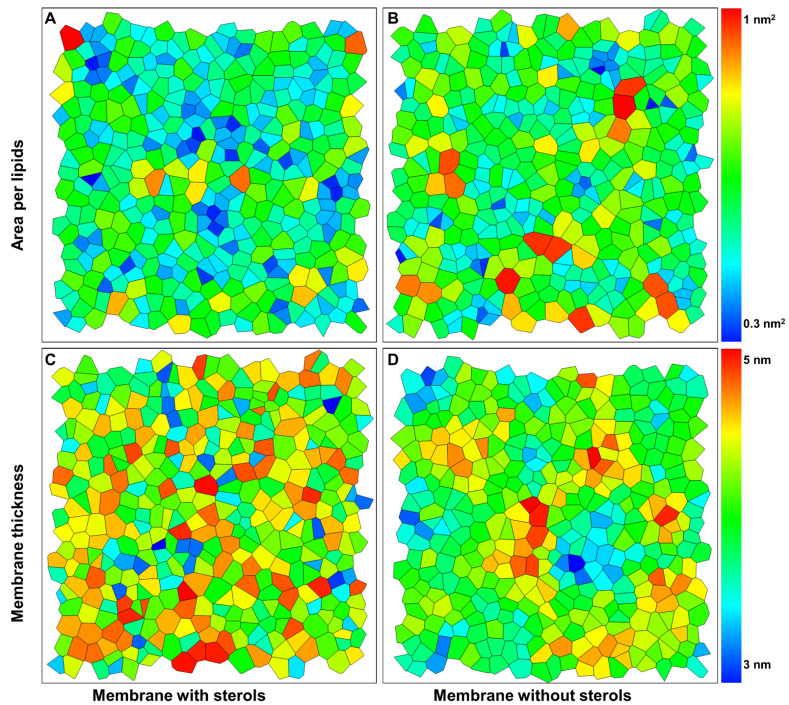
Two-dimensional Voronoi diagrams to characterize the membrane dynamics. (**A**,**B**) Voronoi analysis for x–y projection of the centers of mass of the lipids to obtain the area per lipids in membrane containing and not containing phytosterols. (**C**,**D**) Voronoi analysis for x-y projection of the centers of mass of the lipids to obtain the membrane thickness in model membrane not containing phytosterols.

Area per lipid is an average property and does not contain information about the distribution of lipid molecules. To study the possible changes in the lipid distribution, we performed a Voronoi analysis in two dimensions to obtain the probability distributions for the area per lipid [52]. The areas of the Voronoi cells give the area and thickness distribution of the lipids’ centers of mass (Supplementary Videos S2–S5). In the ordered state, many lipids occupy a very low area per lipid, depicted by the blue color (Figure 6A,B). It is important to note that the high order of membranes containing phytosterols leads to an increase in the membrane thickness represented in the red color (Figure 6C,D).

### 3.6. Lipid–Lipid Interactions Underlying Lateral Domain

**Figure 7:**
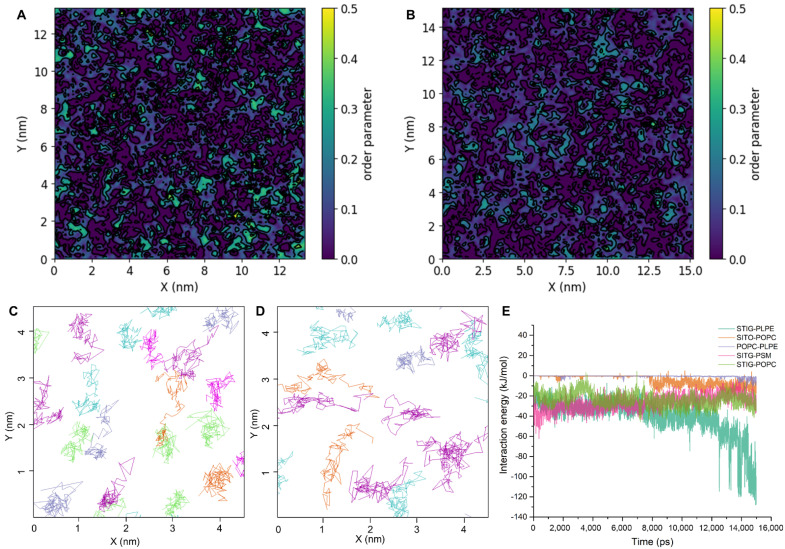
Lipid–lipid interactions are the main driving forces for lipid domain association. (**A**,**B**) Membrane lipid order parameter landscape for model membrane containing and not containing phytosterols, respectively. (**C**,**D**) Lateral trajectories of the center of mass of the lipid molecules in membrane containing and not containing phytosterols over 10 ns MD simulation. Cyan, POPC; purple, PLPE; lime, SITO; magenta, STIG; ice blue, PSM. (**E**) Distributions of pair interaction energies among POPC, PLPE, SITO, STIG and PSM from the simulation time.

Lipid–lipid interactions also play a role in determining the homeostatic dynamics of plasma membranes. To better understand the effect of lipid–lipid interactions on membrane structure, we examined three metrics related to the membrane structure: lipid density, membrane thickness and lipid order. In the system with phytosterols, membrane ordering is significantly higher than those in the system without phytosterols (Figure 7A,B). Lipid order appears to be more sensitive to the presence of phytosterols. As shown in Figure 7A,B, the precise shape and extent of the perturbed areas strongly depend on the presence of phytosterols. The lateral trajectories of the head group of the lipid molecules are plotted in Figure 7C,D. The trajectories clearly show that the lipid molecules are much more mobile in the membrane not containing phytosterols (Figure 7D) than in the normal membrane (Figure 7C), demonstrating that phytosterols contribute to membrane order and fluidity. To quantify the interaction of the lipid components, we further calculated the interaction energy between different lipid pairs (Figure 7E). From the free energy profile, it appears that the STIG and PLPE interaction is more favorable for the formation of the lipid-ordered domain by almost −60 kJ/mol, while the interaction between non-phytosterol lipids is less favorable with the value of ~5 kJ/mol. In line with expectations, the order parameters from the model membranes form two basic clusters: higher ordered regions for the phytosterol-rich domain and lower order regions for the other lipid-rich domain with unsaturated tails.

## 4. Discussion

Membrane microdomains, widely referred to as lipid rafts, are proposed to be liquid-ordered phase domains enriched in sphingolipids and sterol, serving as a central regulatory mechanism in various physiological processes, such as membrane signaling and plant–pathogen interaction [1,2,55]. Although large, micrometer-sized microdomains could be readily observed in artificial membranes [56], attempts to observe the existence of these analogous domains in live cells are still difficult because of the resolution limit of ~10 nm on their size [57,58]. Conventional optical microscopy has a ~200 nm resolution limit and, therefore, does not allow a detailed observation of the membrane microdomain. As plasma membrane microdomains are proposed to be of the order of tens of nanometers, it is important to emphasize that both GP imaging and traditional lifetime imaging cannot image the individual membrane microdomains. The recent state of the art in fluorescence microscopy, so-called super-resolution microscopy or nanoscopy, has opened the door to true nanoscale observation of membrane structure and microdomains [57,59]. There are currently three main types of super-resolution microscopy: stimulated emission depletion (STED), structured illumination microscopy (SIM) and single-molecule localization microscopy (photoactivated localization microscopy, PALM, and stochastic optical reconstruction microscopy, STORM). Single-particle tracking (SPT) notably allows the observation of protein dynamics, protein–lipid interactions in membrane microdomains, providing information on the main forces driving membrane domain formation [58,60,61]. The direct visualization of membrane microdomains in living cells will reveal novel features of the heterogeneities of the plasma membrane.

Probes, such as Laurdan and di-4-ANEPPDHQ, whose spectral properties display a blue shift in response to lipid packing, are widely used to study membrane domains of different lipid phases. However, Laurdan and di-4 harbor long alkane chains, and net charges make it difficult for them to easily penetrate plant cell walls. Organic π-systems end-capped with an electron donor (D) and an electron acceptor (A) are widely known as push–pull chromophores. Some of the fluorophores exhibit improved or additional properties, such as strong solvatochromism [62], high fluorescence quantum yield [63] and color change in different solvent environments [32]. Planarizable push–pull probes provide new types of probes to image lipid packing changes through changes in their fluorescence lifetime. The push–pull probe used in this study is based on pyrene structure [13]. The PA probe used in this study has many advantages compared with Laurdan and di-4, which has a small molecular weight and easily penetrates (<5 min) a plant cell wall at a lower concentration (~100 nM). In this study, we investigated the time-resolved fluorescent parameters to determine lipid structure and dynamics in plant cell membranes, using the ratiometric and solvatochromic membrane probe PA. We first described the GP spectral imaging on living *Arabidopsis* root cells labeled with this higher brightness and photostability PA probe. The GP pseudo-colors for the different parts of the cells indicate differences in the lipid organization of plasma membrane compartments.

To confirm the observed GP variations in the lipid order of the plasma membrane, we carried out fluorescence lifetime imaging experiments on PA in living plant cells. We found that the Lo phase could be discriminated from the Ld phase by the values of its mean fluorescence lifetimes. Specifically, the lifetime is sensitive to the phase localization of the different lipids with a significantly longer lifetime in the Lo domain in comparison to the Ld domain. In the Lo phase, PA displayed a fluorescence lifetime of 6.7 ns, while in the Ld phase, it was 4.3 ns. This result was consistent with the previous study that showed the lifetimes of PA in liposomes (LUVs) were 6.7 ns for the Lo phase and 4.6 ns for the Ld phase, respectively. The large variation in the long lifetime for the wild-type (~7.1 ns) and phytosterol-depleted cells suggests that they are characterized by different local phytosterol compositions (5.8 ns for mβCD treatment and 6.0 ns for *smt2smt3* mutant, respectively). Therefore, sterols are supposed to increase lipid packing and order phase, which cause longer fluorescence lifetimes of the PA probe. Importantly, the use of fluorescence lifetime microscopy allows the quantification of the degree of lipid order and disorder in different compartments inside whole cells. This provides a concentration-independent and sensitive approach [29] with significant advantages compared to intensity-based measurements [64], such as the single-point measurements of diffusion coefficients (e.g., FRAP and FCS).

There has been an increasing number of reports regarding the existence of plasma membrane microdomains in plant cells [4,19,57,65]. However, the detailed understanding of how lipid composition and mobility changes affect the key lipid–lipid interactions and structural changes responsible for the formation of phase separation and microdomain formation still remain as formidable challenges. Despite extensive experimental studies in lipid aggregation and phase behavior, molecular dynamics (MD) simulations at the atomic level provide the highest temporal and spatial resolution of this process, with the potential to capture more detailed and key steps during the formation of microdomains and phase separation [38,66,67]. Several well-known MD simulation packages can conduct dynamics simulations on a single GPU and multiple GPUs with performances that outstrip even the most powerful conventional CPU-based supercomputers [68,69,70]. The remarkable advances in supercomputing and algorithms have enabled us to simulate more complex systems for larger temporal and spatial scales approaching experimental conditions [71,72]. By performing all-atom molecular dynamics simulations of lipid bilayers consisting of five lipid components, we found an increase in lipid order and membrane thickness of the model membrane containing phytosterols, consistent with the FLIM experimental results. In addition, phytosterols can also noticeably reduce the area per lipid from the value deduced from the membrane without phytosterols. Moreover, the free energy profile derived from the MD trajectories showed that STIG and PLPE interaction is more favorable for the formation of the lipid-ordered domain, while the interaction between non-phytosterol lipids is less favorable. Therefore, we can conclude that phytosterols and phosphatidylethanolamines (PEs) play key roles in the formation of membrane microdomains and lipid-ordered phase separation.

## 5. Conclusions

Taken together, our results indicate that the use of the planarizable push–pull probe with lifetime-based imaging allows one to distinguish the Lo phase from the Ld phase with a high contrast in living plant cells. Our MD results have further shown that interactions between STIG and PLPE in the membrane can lead to a high degree of lipid order and phase separation, generating nano-size domains. The combination of optical microscopy and computational microscopy (MD simulation) will significantly increase our knowledge in the interactions of membrane macromolecules with their lipid bilayer environment and expand our understanding of the mechanisms of lipid conformation dynamics, nanodomain formation, phase separation and membrane homeostasis. Further characterization of the membrane phase separation will require techniques with higher spatial resolution, such as single-particle tracking [60,73] and super-resolution microscopy (PALM and STED) [59,74]. We expect our results to also shed light on the mechanism and driving forces of membrane phase separation and membrane nanodomain formation, aiding the characterization and experimental studies in this field.

## Figures and Tables

**Table 1:** Effect of phytosterols on membrane properties.

	Membrane Containing Phytosterols					
	Upside Layer			Downside Layer		
Tested Lipids	Avg. Area (nm^2^)	Avg. Thickness (nm)	Sum. Area (nm^2^)	Avg. Area (nm^2^)	Avg. Thickness (nm)	Sum. Area (nm^2^)
PLPE	0.5	4.21	38.17	0.50	4.22	76.24
POPC	0.52	4.22	58.70	0.52	4.24	46.84
PSM	0.50	4.29	38.71	0.51	4.31	12.12
SITO	0.41	3.85	34.9	0.41	3.86	35.16
STIG	0.41	3.83	9.46	0.42	3.88	9.58
	**Membrane not Containing Phytosterols**					
PLPE	0.58	3.92	96.80	0.58	3.92	95.75
POPC	0.60	3.98	88.06	0.59	3.97	89.72
PSM	0.60	4.04	44.08	0.59	4.08	43.48

## Data Availability

All relevant data have been incorporated in the article and in the associated Supplementary Materials.

## Supplementary Materials

The following are available online at https://www.mdpi.com/article/10.3390/membranes11100747/s1, Video S1: All-atom molecular dynamics simulations of the insertion of PA molecules into a lipid bilayer. Video S2: All-atom molecular dynamics simulations showing the dynamic Voronoi cells of lipid areas in the lipid bilayers containing phytosterols. Video S3: All-atom molecular dynamics simulations showing the dynamic Voronoi cells of lipid thickness in the lipid bilayers containing phytosterols. Video S4: All-atom molecular dynamics simulations showing the dynamic Voronoi cells of lipid areas in the lipid bilayers not containing phytosterols. Video S5: All-atom molecular dynamics simulations showing the dynamic Voronoi cells of lipid thickness in the lipid bilayers not containing phytosterols.
